# 3T magnetic resonance spectroscopy as a powerful diagnostic modality for assessment of thyroid nodules

**DOI:** 10.20945/2359-3997000000069

**Published:** 2018-10-01

**Authors:** Leila Aghaghazvini, Pirouz Pirouzi, Hashem Sharifian, Nasrin Yazdani, Soheil Kooraki, Afsoon Ghadiri, Majid Assadi

**Affiliations:** 1 Tehran University of Medical Sciences Tehran University of Medical Sciences Shariati General Hospital Department of Radiology Tehran Iran Department of Radiology, Shariati General Hospital, Tehran University of Medical Sciences, Tehran, Iran; 2 Tehran University of Medical Sciences Tehran University of Medical Sciences Imam Khomeini Hospital Advanced Diagnostic and Interventional Radiology Research Center Tehran Iran Advanced Diagnostic and Interventional Radiology Research Center, Imam Khomeini Hospital, Tehran University of Medical Sciences, Tehran, Iran; 3 Tehran University of Medical Sciences Tehran University of Medical Sciences Amir Alam General Hospital Department of Radiology Tehran Iran Department of Radiology, Amir Alam General Hospital, Tehran University of Medical Sciences, Tehran, Iran; 4 Tehran University of Medical Sciences Tehran University of Medical Sciences Amir Alam Hospital Otorhinolaryngology Research Center Tehran Iran Otorhinolaryngology Research Center, Amir Alam Hospital, Tehran University of Medical Sciences, Tehran, Iran; 5 Bushehr University of Medical Sciences The Persian Gulf Nuclear Medicine Research Center Bushehr Iran The Persian Gulf Nuclear Medicine Research Center, Bushehr University of Medical Sciences, Bushehr, Iran

**Keywords:** Magnetic resonance spectroscopy, thyroid nodules, thyroid carcinoma, choline

## Abstract

**Objective:**

Magnetic resonance spectroscopy (MRS) is a powerful tool for structural studies of chemical compounds and biomolecules and also documented promising findings as a potential imaging technology in thyroid oncology. This prospective study was to ascertain the clinical significance of 3 Tesla MRS in the evaluation of patients with thyroid nodules (TNs) as an ancillary diagnostic technique for thyroid carcinoma.

**Materials and methods:**

Magnetic resonance spectroscopy at 3T at echo- times (TEs) 136 and 270 ms was carried out on 15 patients with total number of 32 TNs larger than 1 cm^3^, which all were surgically resected. Choline (Chol) to creatine (Cr) ratio was assessed at 136 and 270 TEs on each nodule and a receiver operating characteristic (ROC) curve was used to determine optimal cut-off point. The findings were compared with histopathology of thyroid specimens.

**Results:**

There were 23 benign and 9 malignant lesions (7 papillary and 2 follicular thyroid carcinomas). The mean values of Chol/Cr at 136 and 270 TEs was 2.28 ± 3.65 and 1.52 ± 1.67 respectively and the difference between benign and malignant nodules was only significant at 136 TEs. The study revealed that Chol/ Cr ratio cut-off point of 2.5 best correlates with histopathology results (sensitivity = 75%; specificity = 100%; PPV = 100%; NPV= 92%).

**Conclusion:**

This preliminary study showed that 3T magnetic resonance spectroscopy might be a specific modality for the evaluation of thyroid nodules in differentiation of benign from malignant thyroid tissue. However, a larger series would give much greater confidence that this state-of-the-art technology will worth pursuing in clinical practice.

## INTRODUCTION

Thyroid nodules are quite common and estimated as being clinically presented in 4-7% of the population (1). Early and precise differentiation of malignant and benign nodules stays a remarkable diagnostic problem, but is critical to the patient's management (2). Although there is not a consensus in the method of choice for initial evaluation, it is generally accepted that, adjunct with thyroid function tests, fine- needle aspiration cytology (FNAC), should be the first procedure to be done (3). Nevertheless, with a reported sensitivity of 68-98% (mean 83%) and a specificity of 72-100% (mean 92%); FNAC does not seem to be a perfect diagnostic method for the assessment of thyroid nodules (4-6).

The success of ultrasound as an inexpensive and noninvasive modality in discrimination benign from malignant nodules is presented in several reports (7,8). Although none of the sonographic properties have a reliable and sufficient sensitivity, specificity, positive predictive value (PPV) and negative predictive value (NPV) in ascertaining malignancy of the nodules, the sonographic features of the nodules can assists in selecting the nodules for needle aspiration (7,9). Computed tomography of neck is often restricted by different artifacts (10). Magnetic resonance spectroscopy (MRS) is the only non-invasive modality capable of measuring chemicals/metabolites within the body (11) as result of different magnetic frequency or chemical shifts (11). The concept that malignant cells develop a large number of proton nuclear magnetic resonance (1H NMR) visible molecules leads to potential role of MRS as a diagnostic modality in various cancers (12). It is a powerful tool that has been used the nuclear magnetic resonance in NMR spectroscopy based upon the hydrogen-1 nuclei within the molecules of a matter, for the purpose of ascertaining the structure of its molecules (13).

Literature about MRS application especially using 3T for thyroid lesions is very limited. Most of the studies done initially were ex vivo, either MRS was performed on FNAC specimen or on tissue obtained at surgery (14-16). Most few in vivo studies have been carried out with 1.5 T (12,15,17).

The present study was carried out to assess the findings of MRS of thyroid nodules using a 3T MRI and its correlation with histopathology obtained at surgery.

## MATERIALS AND METHODS

The present study was conducted in a university- affiliated tertiary referral Hospital from September 2010 to February 2013. A total number of 15 patients presenting with thyroid nodule larger than 1 cm^3^ were included in the study. Patients with previous thyroid surgery, known malignant disease or history of radiation were excluded. Moreover, patient's with contraindication for MRI such as claustrophobia and also being pace maker were excluded.

All MRI examinations were performed on a 3 Tesla MR unit (Magnetom Avanto; Siemens, Erlangen, Germany) with gradient strength of 33 mTs. Patients were positioned in supine position and were instructed not to swallow or move during the examination. Circularly polarized surface coil was placed over the neck. Fast scout scan in sagittal, axial and coronal planes was obtained. After localizing the lesion, the voxel was placed on the lesion and position checked in all three planes. The scan technique used was PRESS, single voxel technique. The sequence parameters employed with sampling numbers 512, averaging 16 and average scan time of 4.55 min. It was followed by water suppression pulses to be followed by data acquisition. Choline (Chol) to creatine (Cr) ratio was assessed at 136 and 270 TEs on each nodule. Image analyses were assessed by two radiologists and discrepant results were resolved by consensus and these findings were compared with the results of histopathological studies as gold standard test.

All patients had previously undergone ultrasound examination, FNAC and thyroid hormone measurements and also had previously been selected for surgery on the basis of their clinical and paraclinical results.

The study complies with the declaration of Helsinki and was approved by the institutional ethics committee of Tehran University of Medical Sciences, and all patients gave written informed consent.

### Statistical analysis

The distribution of parameters was evaluated using probability plots and the Shapiro-Wilk test. Continuous variables are presented as mean ± SD, and categorical values as the absolute values and percentages. Fisher exact and chi-squared tests were applied to compare the qualitative variables and also T-test and *Mann-Whitney U test* between the quantitative variables in groups. The sensitivity, specificity, positive and negative predictive and also efficiency of values of each setting was acquired based on the definitive diagnosis established through histopathology. A receiver operating characteristic (ROC) curve analysis was done to determine optimal cut-off point for the discrimination of malignant from benign nodules. Cohen's kappa coefficient (κ) was applied inter-rater agreement of variables. Statistical analysis was performed using an IBM computer and PASW software, version 22.0 (SPSS, Inc., Chicago, USA).

## RESULTS

A total of 15 patients (11 women, 4 men; mean age 39.5 ± 12.3 years, range 20-50) were included in this study. The total number of thyroid nodules was 32; 15 nodules were in the right lobe; 13 nodules in the left lobe and 4 nodules in the isthmus. Histological findings were malignant in 9 cases (seven cases of papillary carcinoma and two cases follicular carcinoma) and benign in 23 cases (Table 1). The volume of thyroid nodules was 11.67 cc; there was no significant difference in volume between malignant and benign nodules (p value > 0.05).

**Table 1:** Histopathology of thyroid nodules of the included patients

	Frequency	Percent
Coloid cyst	4	12.5
Coloid nodule	7	21.9
Hashimoto	2	6.3
MNG	6	18.8
MNG with lymphocytic thyroiditis	2	6.3
Nodular hyperplasia with cystic degeneration	2	6.3
Follicular thyroid carcinoma	2	6.2
Papillary thyroid carcinoma	7	21.9

MNG: multinodular goiter.

In term of metabolite profile semi quantitatively, significant choline peak was seen only in 7 cases (malignant = 7, benign = 0). The mean values of Chol/Cr at 136 and 270 TEs was 2.28 ± 3.65 and 1.52 ± 1.67 respectively and the difference between benign and malignant nodules was only significant at 136 TEs (p value < 0.05) (Table 2). Seven malignant cases showed significant choline peak at 3.22 ppm whereas 2 out of 9 malignant cases failed to show it. Among 23 benign lesions, none showed choline peak at 3.22 ppm.

**Table 2:** Correlation between mean of different variables and pathology

	Pathology	N	Mean ± SD	*P* value
Chol/cr 136 ms	Benign	23	0.80 ± 0.60	0.018
	Malignant	9	6.60 ± 5.30	
Chol/cr 270 ms	Benign	10	0.80 ± 0.63	0.088
	Malignant	6	2.71 ± 2.20	

Correlation of spectroscopic findings with pathologic diagnosis was presented in Table 3.

**Table 3:** Correlation of spectroscopic findings with pathologic diagnosis

Cho/cr	Number	Cut off 1.5		Cut off 2.5	
		TE 136	TE 270	TE 136	TE 270
Colloid cyst	4	0	0	0	0
Colloid nodule	7	1 of 7 (2.32)	0	0	0
Hashimoto	2	0	0	0	0
MNG	8	1 of 8 (1.67)	0	0	0
Nodular hyperplasia	2	1 of 2 (2.22)	1of 2 (2.32)	0	0
FCC	2	0	0	0	0
PTC	7	7 of 7	4 of 4	7 of 7	3 of 4 (2.39)

**Figure 1:**
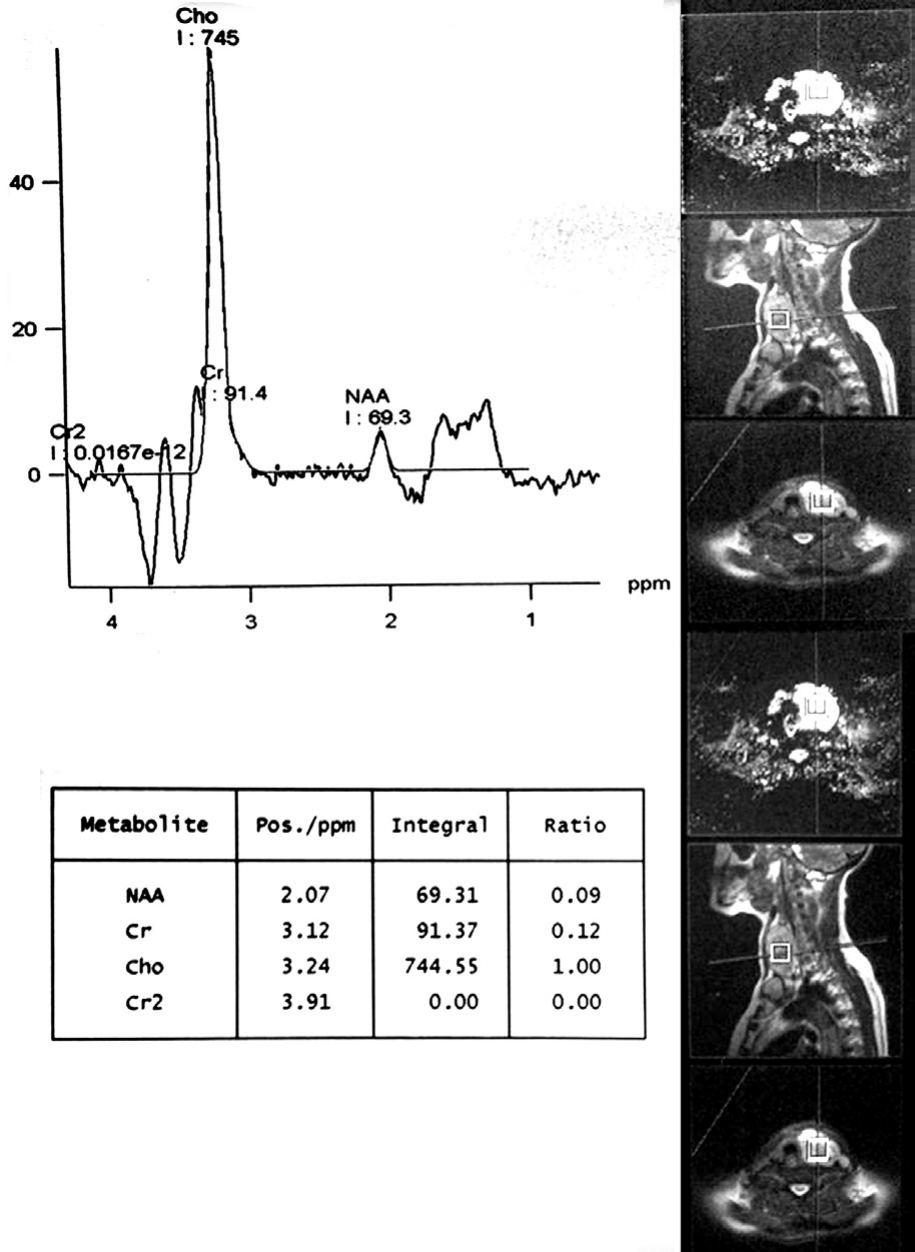
Graph of a malignant thyroid nodule with a very high choline (Chol) to creatine (Cr) ratio on MRS.

**Figure 2:**
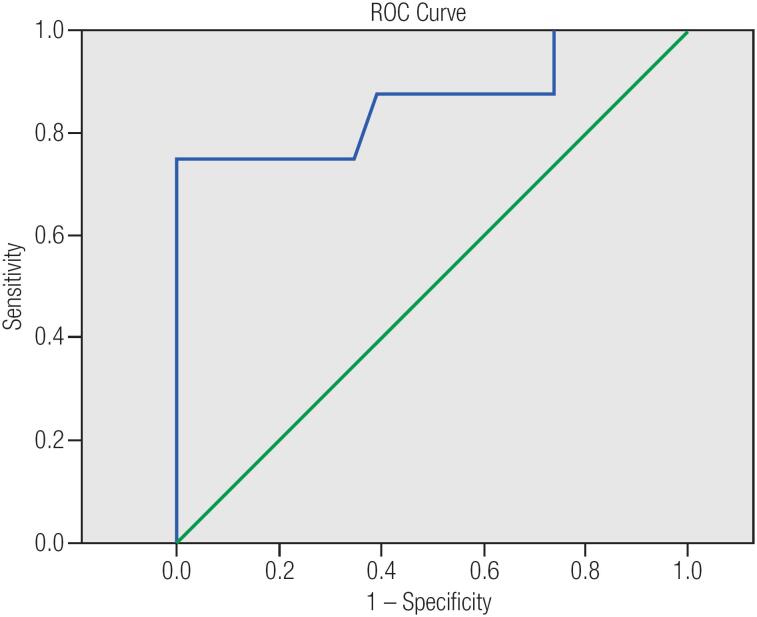
Receiver operating characteristic curve analysis for the prediction of malignant thyroid nodules based on choline (Chol) to creatine (Cr) ratio at 136 TE.

The area under the ROC curve (AUC) of Chol/Cr was significant at 136 TEs (0.86, p value = 0.00) while was not statistically at 270 TEs (0.78, p value = 0.68). Roc curve study revealed that Chol/Cr ratio at 136 TEs with a cut-off point of 2.5 best correlates with histopathology results (sensitivity = 75%; specificity = 100%; PPV = 100%; NPV= 92%) (Figures 1 and 2, Table 4).

**Table 4:** Diagnostic values for Chol/Cr in cut off points at 136 TEs

Cut off	0.35	0.85	1.5	2.5
Sensitivity	1 (95% CI: 0.63 – 1)	0.88 (95% CI: 0.47 – 0.99)	0.75 (95% CI: 0.35 – 0.97)	0.75 (95% CI: 0.35 – 0.97)
Specificity	0.26 (95% CI: 0.10 – 0.48)	0.61 (95% CI: 0.39 – 0.80)	0.87 (95% CI: 0.66 – 0.97)	1 (95% CI: 0.85 – 1)
Efficiency	0.45 (95% CI: 0.27 – 0.64)	0.68 (95% CI: 0.49 – 0.83)	0.68 (95% CI: 0.49 – 0.83)	0.94 (95% CI: 0.79 – 0.99)
Positive Predictive value	0.32(95% CI: 0.15 – 0.56)	0.44 (95% CI: 0.20 – 0.70)	0.44 (95% CI: 0.19 – 0.70)	1.00 (95% CI: 0.45 – 1.00)
Negative Predictive value	1 (95% CI: 0.54 – 1)	0.93 (95% CI: 0.68 – 0.99)	0.93 (95% CI: 0.68 – 0.99)	0.92 (95% CI: 0.74 – 0.99)
Likelihood ratio of positive test	1.35 (95% CI: 1.06 – 1.72)	2.24 (95% CI: 1.26 – 3.97)	2.27 (95% CI: 1.26 – 3.97)	–
Likelihood ratio of negative test	–	4.87 (95% CI: 0.76 – 31.36)	4.87 (95% CI: 0.76 – 31.36)	4.00 (95% CI: 1.20 – 13.28)
Cohen's kappa coefficient (κ)	0.15 (95% CI: 0.01 – 0.30)	0.36(95% CI: 0.086 – 0.64)	0.36 (95% CI: 0.06 – 0.64)	0.82 (95% CI: 0.58 – 1.06)

## DISCUSSION

The study depicted that the findings of 3T MRS of thyroid nodules correlated with histopathology obtained surgically.

In term of imaging, ultrasonography, as a main modality in the management of patients with thyroid nodules, has some very useful discriminatory values in distinguishing benign from malignant lesion (4,7,18).

Though computed tomography and MR imaging studies allow a rapid and accurate assessment of the size of a goiter, its extension into the mediastinum, and its relationship to and impingement upon major structures with in the chest and neck, their accuracy is not high enough to different benign from malignant lesions (19).

Parallel in anatomical and functional changes, in addition to histologic and cytologic alterations in malignancies, metabolic processes at the cellular level are also identified to trace changes. These metabolic changes can be discovered and quantified using a state of the art technique acknowledged as magnetic resonance spectroscopy (MRS) which has recently gained interest as a potential cancer imaging technology e.g. in thyroid cancer. Literature about MRS of in vivo evaluation of thyroid lesions is very limited (17,20-22).

In a study, 1HMRS was carried out over tissue obtained at the time of surgery from 53 patients undergoing partial or total thyroidectomy for thyroid nodule (20). When compared with histological diagnosis, 1HMRS distinguished normal thyroid tissue from invasive papillary, anaplastic and medullary carcinoma with *P* values of < 0.001 with negative predictive value 100% and specificity of 52% (20).

Gupta and cols. studied 1.5T MRS in 25 patients with solitary thyroid nodule and they depicted a sensitivity and specificity of 100% and 94.11% respectively (21). They also had worked previously on 26 patients using 1.5T MRS and demonstrated sensitivity of 100% and specificity of 88.88% (22). King and cols. also worked 1HMRS in 13 patients of thyroid nodules with larger than 1 cm and demonstrated sensitivity of 87% and specificity of 100% (17).

In the current study, 3T MRS was carried out on 32 thyroid nodules in which the Chol/Cr ratio cut-off point of 2.5 best correlates with histopathology results (sensitivity = 75%; specificity = 100%; PPV = 100%; NPV = 92%).

In this study, 2 out of 9 malignant cases failed to delineate the choline peak at 3.22 ppm which most probably because of small size of follicular cell carcinoma lesions.

In addition, 3 benign thyroid nodules showed Chol/Cr more than 1.5 but less than 2.5 which could be due to hyperplastic foci as result of increased hypercelluraity as shown in 2 hyperplastic nodules in prior study (22).

In the current study, the sensitivity (75%) is less than two main prior studies (King and cols., 87%; Gupta and cols., 100%) while the specificity is comparable to two studies. It could be explained by two small follicular cancer lesions in our study.

In term of metabolite profile assessment, choline peak at 3.22 ppm is predominantly due to glycerophosphocholine and glycerophosphoethanolamine that form phospholipids of the cell membranes (23). The choline content rises in malignancy because of rapid multiplication and proliferation of cells (23). Height of choline peak depends on amount and nature of tissue under voxel. The creatinine peak indicates energy state of the cell (14,24).

In total, the numbers of cases especially cancers in this series is low and further studies with a larger series would give much greater confidence that the technique was worth pursuing in clinical practice.

In conclusion, magnetic resonance spectroscopy is a feasible option with promising results and it provided 100% specificity and 100% PPV in discrimination of benign from malignant thyroid nodules. Therefore, it can be complementary to other diagnostic techniques in patients with thyroid nodules; however, further exploration especially considering large number of patients with different thyroid nodules sizes is needed to validate its clinical role.
